# The TiltMeter app is a novel and accurate measurement tool for the weight bearing lunge test

**DOI:** 10.1186/1757-1146-6-S1-P17

**Published:** 2013-05-31

**Authors:** Cylie Williams, Antoni Caserta, Terry Haines

**Affiliations:** 1 Southern Health Allied Health Clinical Research Department, Cheltenham, VIC 3192 , Australia; 2 Monash University, Department of Physiotherapy, Frankston, VIC, 3199, Australia; 3 Cardina Casey Community Health Service, Cranbourne, VIC, 3197, Australia

## Background

The weight bearing lunge test is increasing being used by health care clinicians who treat lower limb and foot pathology. This measure is commonly established accurately and reliably with the use of expensive equipment. This study aimed to compare the digital inclinometer with a free app, TiltMeter on an Apple iPhone.

## Methods

Allied health practitioners were recruited as participants from the workplace. A preconditioning stretch was conducted and the ankle range of motion was established with the weight bearing lunge test position with firstly the leg straight and secondly with the knee bent. The measurement device and participant order was randomised.

## Results

The intra-rater reliability and inter-rater reliability for the devices and in both positions were all over ICC 0.8 except for one intra-rater measure (Digital inclinometer, novice, ICC 0.65). The inter-rater reliability between the digital inclinometer and the TiltMeter was near perfect, ICC 0.96 (CI: 0.898–0.983); Concurrent validity ICC between the two devices was 0.83 (CI: -0.740–0.445).

## Conclusion

The use of the TiltMeter app on the iPhone is a reliable and inexpensive tool to measure the available ankle range of motion. Health practitioners should use caution in applying these findings to other smart phone equipment if surface areas are not comparable.

